# Geometric morphometric methods for identification of oyster species based on morphology

**DOI:** 10.3897/BDJ.12.e115019

**Published:** 2024-02-26

**Authors:** Qian Liu, Yuepeng Guo, Yanzhuo Yang, Junxia Mao, Xubo Wang, Haijiao Liu, Ying Tian, Zhenlin Hao

**Affiliations:** 1 Dalian Ocean University, Dalian, China Dalian Ocean University Dalian China; 2 Dalian Changhaiyide Aquaculture Company,Dalian,China, Dalian, China Dalian Changhaiyide Aquaculture Company,Dalian,China Dalian China; 3 Dalian Shell Museum, Dalian, China Dalian Shell Museum Dalian China

**Keywords:** traditional morphometrics, geometric morphometrics, Pacific oyster

## Abstract

Both genetic and environmental factors affect the morphology of oysters. Molecular identification is currently the primary means of species identification, but it is inconvenient and costly. In this research, we evaluated the effectiveness of geometric morphometric (GM) techniques in distinguishing between two oyster species, *Crassostreagigas* and *C.ariakensis*. We used traditional morphometric and GM methods, including principal component analysis (PCA), thin-plate spline analysis (TPS) and canonical variable analysis (CVA), to identify specific features that distinguish the two species. We found that differences in shape can be visualised using GM methods. The Procrustes analysis revealed significant differences in shell morphology between *C.gigas* and *C.ariakensis*. The shells of *C.ariakensis* are more prominent at the widest point and are more scattered and have a greater variety of shapes. The shells of *C.gigas* are more oval in shape. PCA results indicated that PC1 explained 45.22%, PC2 explained 22.09% and PC3 explained 10.98% of the variation between the two species, which suggests that the main morphological differences are concentrated in these three principal components. Combining the TPS analysis function plots showed that the shell shape of *C.ariakensis* is mainly elongated and spindle-shaped, whereas the shell shape of *C.gigas* is more oval. The CVA results showed that the classification rate for the two species reached 100% which means that *C.ariakensis* and *C.gigas* have distinct differences in shell morphology and can be completely separated, based on morphological characteristics. Through these methods, a more comprehensive understanding of the morphological characteristics of different oyster populations can be obtained, providing a reference for oyster classification and identification.

## Introduction

Oysters belong to the Phylum Mollusca, Class Bivalvia, Order Pterioida and Family Ostreidae (Zhang and Lou 1956, Ruppert 2013, Bayne et al. 2017). Over 100 species of oysters have been discovered to date (Lindberg and Ponder 2008, Bayne et al. 2017). They have a worldwide distribution and are an important marine biological resource (Bieler et al. 2010, Wu et al. 2011, Salvi et al. 2014). They are also important aquaculture species both domestically and internationally (Dong et al. 2004, Wijsman et al. 2018, Bayne et al. 2019). In coastal areas of China, 30 species of oysters had been reported by Xu (1997) and the report in 2008 listed 23 species (Xu and Zhang 2008). Recent research indicates that a total of 37 species of oysters have been discovered in China to date (Li et al. 2017, Guo et al. 2018, Hu et al. 2019, Cui et al. 2021).

Due to the susceptibility of oyster shells to environmental changes, oyster classification has always been controversial. The continuous study of oyster classification has resulted in a relatively mature oyster classification system (Que et al. 2003). By sequencing and analysing the oyster genome, the classification position and relationship of oysters has become more accurate, providing new means for oyster classification research (Wang et al. 2007). However, during practical production and aquaculture processes, the application of molecular techniques is not feasible for non-destructive classification of large numbers of specimens.

Morphometrics is a method for studying trait variation and its covariance with other variables (Rohlf and Slice 1990). Currently, geometric morphometric (GM) methods are widely used in medicine (Du and Lu 2006), botany (Su et al. 2021) and biological classification (Minton et al. 2008, Perez 2011, Miller 2016). Shu et al. (2022) used GM to analyse the morphological differences of eight scallop species in China. Jiang et al. (2019) successfully used GM to identify different geographic populations of the Chinese mitten crab (*Eriocheirsinensis*). Al-Kandari et al. (2021) stated that the current understanding of oyster evolutionary diversity is incomplete and that molecular data are crucial for oyster classification and identification. Molecular identification is currently the primary means of species identification, while it is inconvenient and costly. Comparing GM and molecular identification results is important for improving the accuracy of species classification, which would be highly valuable for oyster species classification.

The methods used in this study are traditional morphometric measurements, as well as multivariate linear analysis, principle component analysis (PCA), thin-plate spline analysis (TPS) and canonical variable analysis (CVA) to analyse the morphological differences between two oyster species (*Crassostreagigas* and *C.ariakensis*).

## Material and Methods

### Sample collection

**Figure 1.**
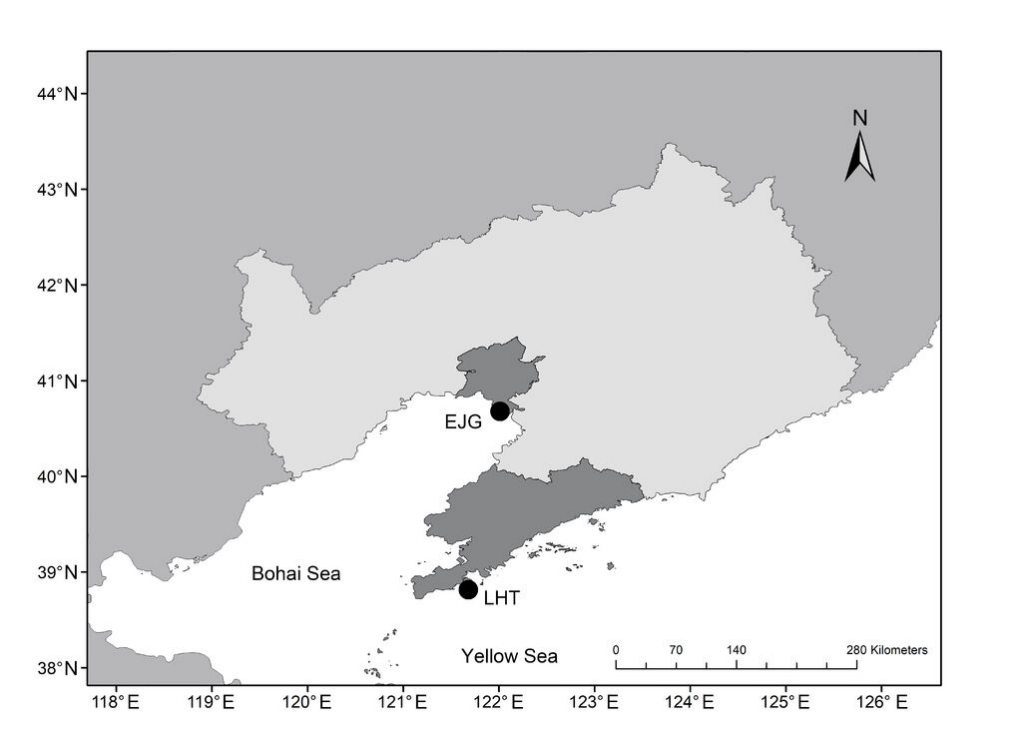
Location of sampling.

In November 2022, oysters were randomly collected from two sites: Erjiegou (40.81°N, 121.97°E) and Laohutan (38.90°N, 121.67°E) in Liaoning Province, China (Fig. 1). A total of 57 (31 samples from Erjiegou and 26 samples from Laohutan) oysters were transported to the Key Laboratory of Mariculture and Stock Enhancement in the Northern Sea Area, Ministry of Agriculture and Rural Affairs, Dalian Ocean University for temporary culture. After removing surface attachments, the oysters were dissected and measured.

### DNA identification

Genomic DNA was extracted from the tissues of six different oyster specimens, with three samples randomly selected from each locality, using the Ezup Column Animal Tissue Genomic DNA kit (Sangon Biotech, Shanghai, China) and the quality of the extracted DNA was checked using 1% agarose gel electrophoresis (Hu et al. 2019, Cui et al. 2021). The primers for the 16S rRNA gene were designed using Primer Premier 5 software. The primer sequences were as follows: 16SF (5′-CGCCTGTTTATCAAAAACAT-3′) and 16SR (5′-CCGGTCTGAACTCAGATCACGT-3′). PCR amplification was performed using the following reaction system: 1.0 µl of DNA template (50 ng/µl), 1.0 µl of 16SF primer (10 µmol/l), 1.0 µl of 16SR primer (10 µmol/l), 2.0 µl of dNTP (2.5 mmol/l), 2.0 µl of 10× buffer, 1.0 µl of Mg^2+^ (25 mmol/l) and 0.1 µl of Taq (5 U/µl) and ultrapure water was added to adjust the final volume to 20 µl.

The PCR programme was as follows: pre-denaturation at 94°C for 3 min; denaturation at 94°C for 30 s, annealing at 52°C for 30 s and extension at 72°C for 1 min, repeated for 35 cycles; and a final extension at 72°C for 10 min, followed by storage at 4°C. PCR products were checked by electrophoresis. The PCR product was preliminarily detected using a gel imaging system. The DNA sequence was obtained using sequence analysis software and it was manually corrected, based on the sequence and peak charts. Amplicon purification and cycle sequencing were conducted by Sangon Biotechnology Co., Ltd., Shanghai, China.

### Traditional morphological measurements

After removing surface attachments, electronic calipers (accurate to 0.01 mm) were used to measure the shell height (SH), shell length (SL) and shell width (SW) of each oyster shell. An electronic scale (accurate to 0.01 g) was used to measure the wet weight (WW), shell weight (SM) and soft tissue weight (ST) of each oyster. The data obtained from Laohutan and Erjiegou were analysed for correlation between each trait using the Pearson correlation coefficient in SPSS 26.0 software (IBM, Armonk, NY, USA). Multiple linear regression analyses were performed using oyster individual SL, SW and SH to establish the optimal multiple linear regression equation between morphological traits and quality traits in order to identify differences in oyster population morphology between the two sampling sites (Suppl. material 1).For this study, we examined 31 specimens of *C.gigas* and 26 specimens of *C.ariakensis*.

### GM measurements

GM image acquisition was conducted as follows: After dissecting the oysters to remove the soft tissue, the right shell of each oyster was photographed with a digital camera (Canon G12, Tokyo, Japan) to capture a two-dimensional image from which data were collected. The shells were photographed in the same orientation such that the vertical line of the umbo was on the Y-axis and the disc on the same plane was parallel to the camera at the same distance. To reduce accidental errors, the photography and subsequent digitisation work were completed by one person (Bai et al. 2014).

### Normalisation of data processing

**Figure 2.**
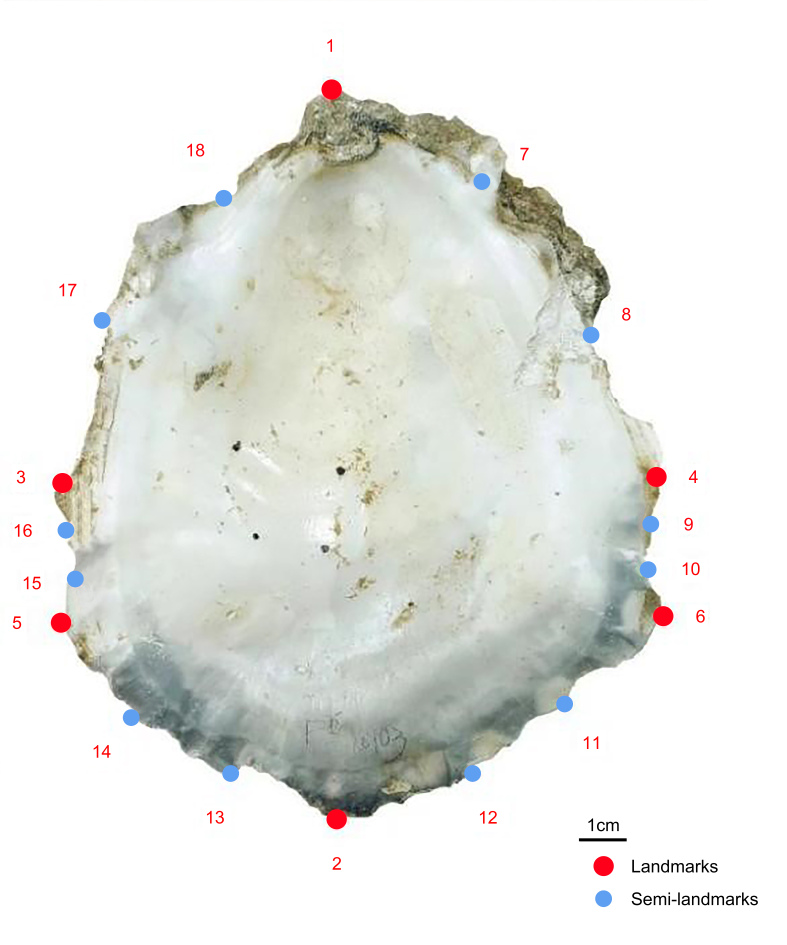
Landmarks and semi-landmarks on the oyster shell. Red dots to represent landmarks and blue dots to represent semi-landmark.

We used landmarks and semi-landmarks to mark and collect data from the two-dimensional images of oyster shells from two different regions. The selection of landmarks is required to reflect the morphological differences of the research objects as well as homology amongst the samples. Semi-landmarks are used to determine the overall outline of the research object more precisely. We selected 18 points, consisting of 1–6 landmarks as biological feature points of oysters and 7–18 semi-landmarks as semi-landmarks along the contour of the oyster shell. These landmarks are as follows: (1) shell apex; (2) posterior margin; (3) the widest point on the left side of the adductor muscle; (4) the widest point on the right side of the adductor muscle; (5) the widest point on the left side of the shell; (6) the widest point on the right side of the shell; (7–8) three equal points from the shell apex to the widest point on the right side of adductor muscle; (9–10) three equal points from the widest point on the right side of the adductor muscle to the widest point on the right side of the shell; (11–12) three equal points from the widest point on the right side of the shell to the posterior margin; (13–14) three equal points from the posterior margin to the widest point on the left side of the shell; (15–16) three equal points from the widest point on the left side of the shell to the widest point on the left side of the shell adductor muscle; and (17–18) three equal points from the widest point on the left side of the shell adductor muscle to the shell apex. The figure below shows the landmarks and semi-landmarks (Valladares et al. 2010) (Fig. 2).

The morphological data for the two oyster species were analysed using Generalised Procrustes Analysis (GPA) in the software Past v.3.24 (Hammer et al. 2001). The analysis removed the effects of non-shell morphological differences caused by differences in shooting angles and landmark selection positions, sizes and orientations (Wang et al. 2017). Subsequently, the data transformed by GPA were subjected to PCA and CVA.

## Results

### DNA indentification

The 16SrRNA gene sequences of six samples from the two sampling sites were determined and the sequencing results were aligned and compared using MEGA v.7.0.26. The obtained haplotype sequences were compared with the relevant sequences downloaded from NCBI. Haplotypes for 16S rRNA sequences were identiﬁed using DnaSP 5software (Librado and Rozas 2009). The Erjiegou oyster population was identified as *C.ariakensis* (accession number: OR598760/OR598761/OR598762) and the Laohutan oyster population was identified as *C.gigas* (accession number: OR598763/OR598764/OR598765) (Suppl. material 2).

### Traditional morphological measurements

**Figure 3.**
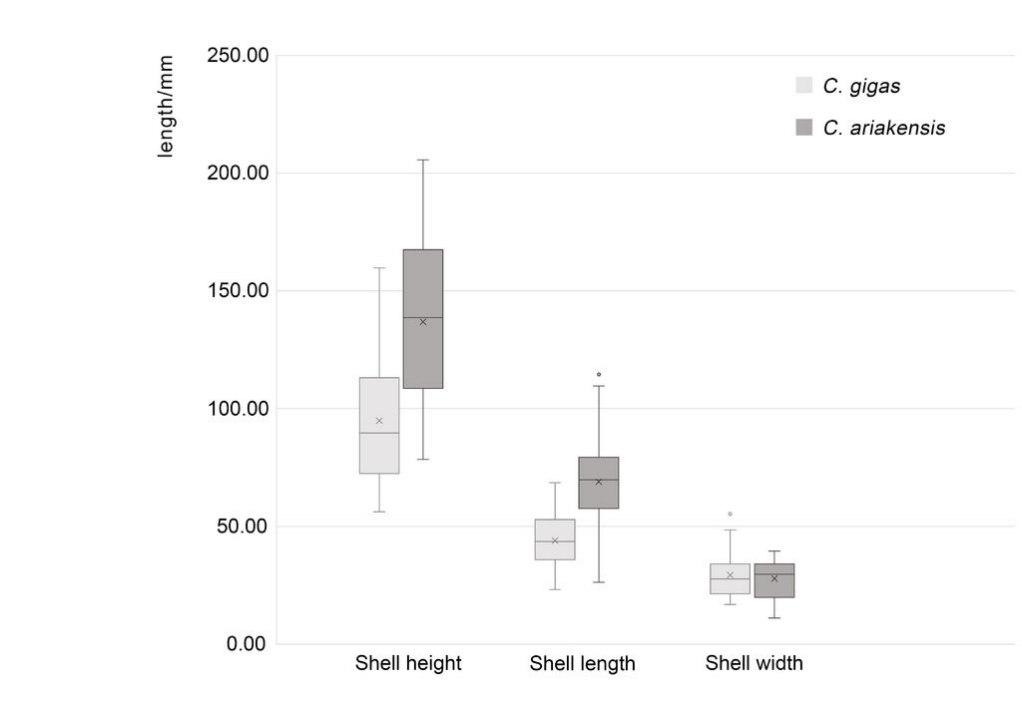
Box plot of morphometric measurements for the two oyster species.

The morphological data for the two oyster species are shown in Table 1 and Fig. 3. For *C.gigas*, SH ranged from 56.19 to 159.78 mm (mean, 94.92 ± 27.91 mm), SL ranged from 23.19 to 68.55 mm (mean, 43.96 ± 11.15 mm) and SW ranged from 16.79 to 55.29 mm (mean, 29.33 ± 9.60 mm). The coefficients of variation were 29%, 25% and 33% for SH, SL and SW, respectively. For *C.ariakensis*, SH ranged from 78.49 to 205.64 mm (mean, 136.89 ± 32.95 mm), SL ranged from 26.28 to 114.53 mm (mean, 69.01 ± 18.63 mm) and SW ranged from 11.12 to 39.57 mm (mean, 27.85 ± 8.17 mm). The coefficients of variation were 24.07%, 26.99% and 29.32% for SH, SL and SW, respectively (Suppl. material 1).

### GM measurements

#### GPA

**Figure 4.**
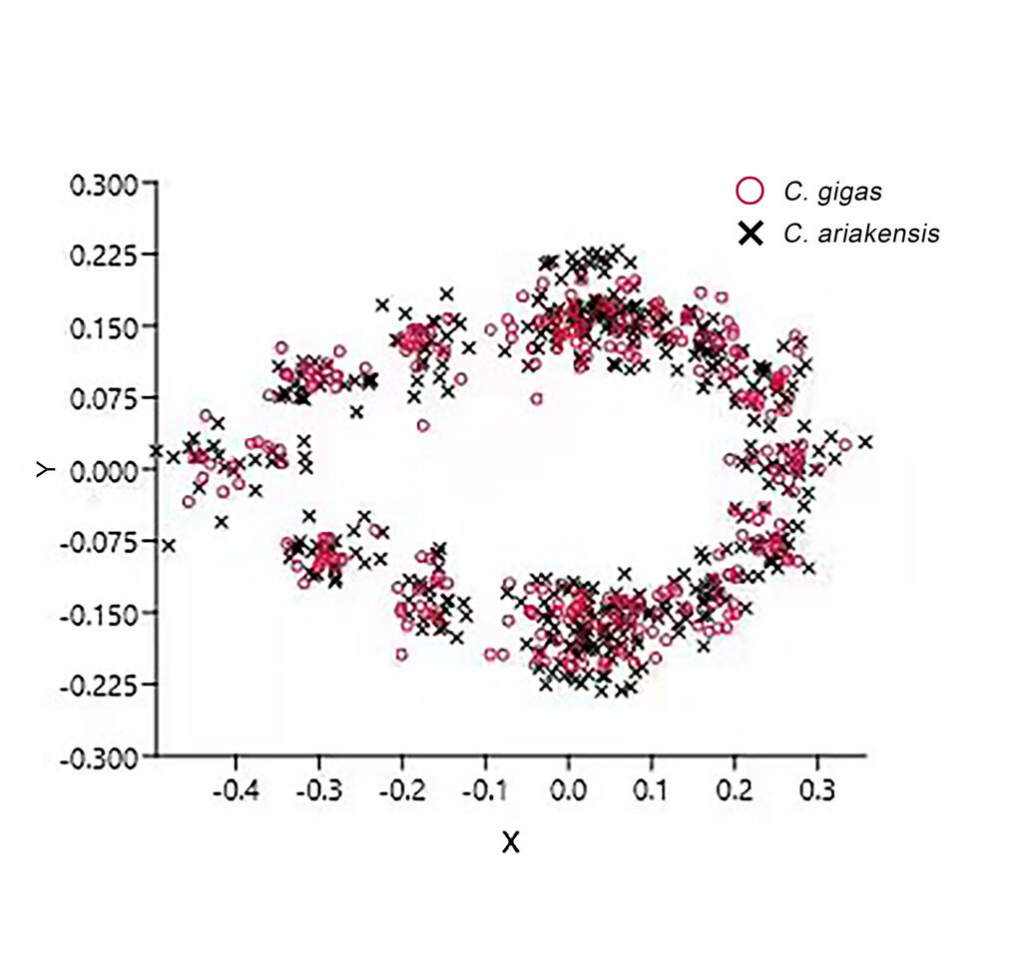
Superimposed map of the GPA of the two oyster species.

GPA was performed on the data using the software Past v.3.24 and the resulting morphological traits of the two groups were quantified and projected on to a coordinate system to obtain a GPA overlay plot (Fig. 4) for the two oyster species. *C.ariakensis* is concentrated in the widest part of the adductor muscle and the widest part of the shell, with the widest part of the shell more prominent compared to that of *C.gigas*. Some points in *C.ariakensis* are dispersed at overlapping points. Most of the shell contour points of *C.gigas* are located inside those of *C.ariakensis*, with few scattered points and a more even distribution overall. The non-morphological factors that may have influenced the results were removed during the analysis (Suppl. material 3).

#### PCA and TPS

**Figure 5.**
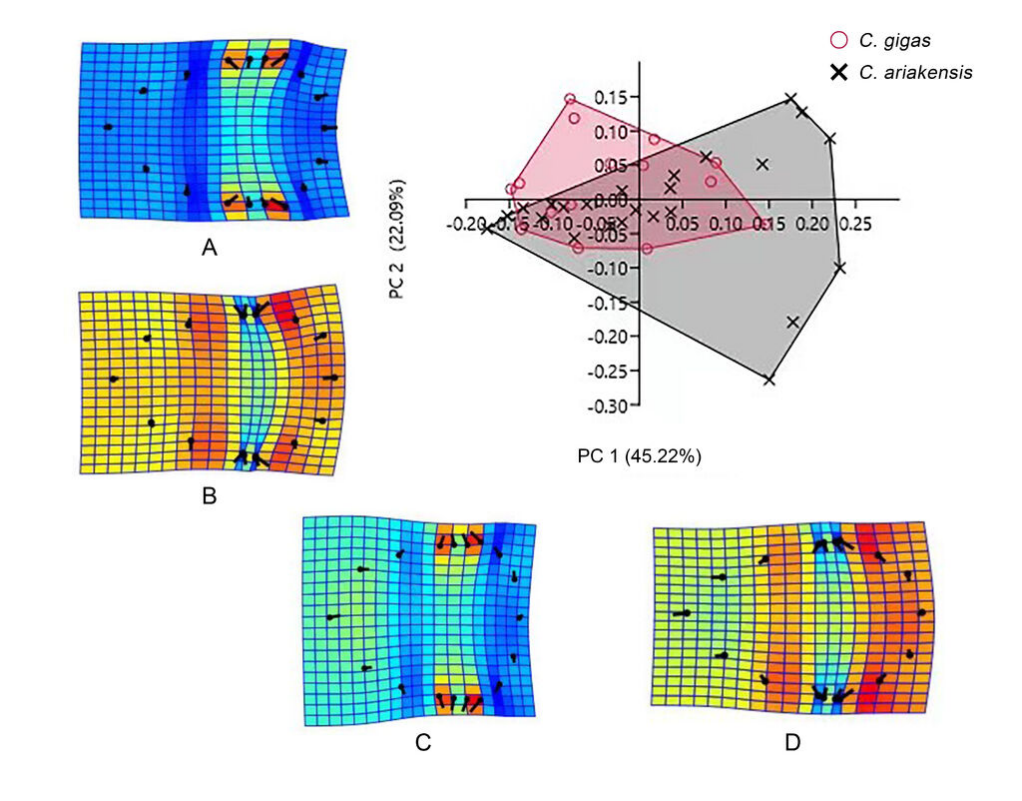
PCA and TPS analysis of the two oyster species. The four deformation maps depicted by a thin-plate spline show the differences between the average shape and the extreme case of each PC.

In the PCA analysis, the first three PCs together account for 78.29% of the total variance and explain the major morphological differences between *C.ariakensis* and *C.gigas*. PC1 contributes 45.22%, PC2 contributes 22.09% and PC3 contributes 10.98% to the total variance. PC1 and PC2, which together account for 85.98% of the total variance, were used as the x and y axes to create the scatter plot (Fig. 5). Along the positive half-axis of PC1, the shell is elongated outwards at its widest point, forming a spindle shape. Along the negative half-axis of PC1, the shell narrows inwards at its widest point, exhibiting the opposite morphology. Along the positive half-axis of PC2, the shell is elongated outwards at its widest point, whereas the ventral margin of the shell contracts inwards. Along the negative half-axis of PC2, the shell narrows inwards at its widest point, but the ventral margin expands outwards. *C.ariakensis* is mainly distributed in the first and third quadrants, displaying a more elongated spindle-shaped morphology. *C.gigas* is predominantly distributed in the second and third quadrants, exhibiting a more oval-shaped morphology. These results indicate significant morphological differences between *C.ariakensis* and *C.gigas*. The oysters in both species are mainly concentrated between PC1 axis –0.15 to 0.15 and PC2 axis –0.07 to 0.15, but the distribution of *C.gigas* is more concentrated, with lower variability (Fig. 5). *C.ariakensis* exhibits a larger distribution and greater variability.

TPS are deformations of a square grid, based on the differences in landmark positions between two shapes. Combining the TPS function images (A–D) revealed that the main variable points along the PC1 axis were 1, 3, 4, 5, 6, 7, 8, 9, 10, 11, 12, 13, 14, 15 and 16, while the main variable points along the PC2 axis were 2, 3, 4, 5, 6, 9, 10, 12, 13, 15 and 16. When the abscissa of the variable points changes in the positive direction along the PC1 axis, there is a tendency for the outermost edge of the shell to grow outwards and the widest part of the shell body expands outwards. When the PC1 axis changes in the negative direction, the shell body becomes shorter and the posterior edge of the shell contracts inwards. When the ordinate of the variable points changes in the positive direction along the PC2 axis, the posterior edge of the shell contracts inwards and the widest part of the shell body expands outwards. When the ordinate of the variable points changes in the negative direction along the PC2 axis, the posterior edge of the shell expands outwards, the widest part of the shell body contracts inwards and the part between the widest part of the shell body and the widest part of the oyster adductor muscle grows narrower. The distribution of the *C.ariakensis* in the positive direction of PC1 and the negative direction of PC2 in the figure is wider than that of the *C.gigas* population, indicating that *C.ariakensis* has a greater degree of variation in the posterior edge of the shell and the widest part of the shell body.

#### CVA

**Figure 6.**
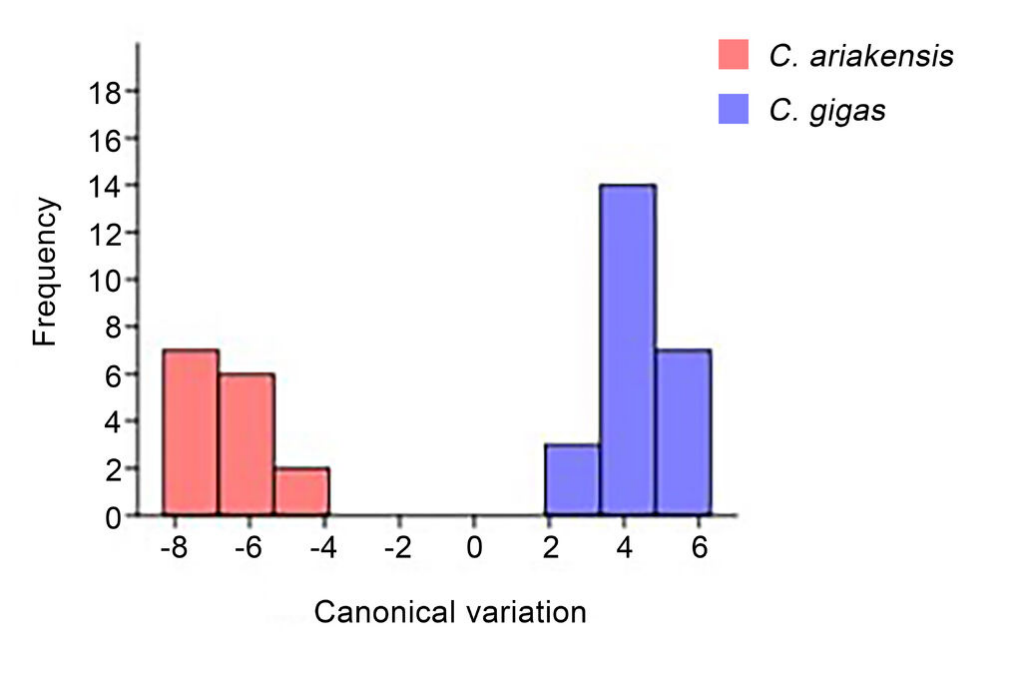
Canonical Variate Analysis (CVA) diagram of shell shapes in the two oyster species.

The results of the CVA, based on Mahalanobis distances and Procrustes distances that were calculated using the within-group covariance matrix and the between-group covariance matrix, respectively, were used to test for significant differences between predefined groups (developmental stage, sex, species) and to evaluate the reliability of classification. The Mahalanobis distance is used to represent the morphological differences between an individual and other individuals within the same population, while the Procrustes distance is used to represent the morphological differences between different groups (Fig. 6).

Figure 6 shows the results of the CVA for *C.gigas* and *C.ariakensis*. The data were imported into PAST software and the typical variable analysis histogram was plotted using morphological discriminant variables as the abscissa and sample frequency as the ordinate *C.gigas* and *C.ariakensis* are completely separated, with a discrimination rate of 100%, indicating that they can be completely separated and are two different species.

## Discussion

Oysters have high morphological plasticity and easily change their shell characteristics, based on the environment. Traditional classification is mainly based on shell morphology and anatomical structure, which can lead to confusion about taxonomic identification (Littlewood 1994). Zhang and Lou (1956) used the classification system proposed by Hirase (1930) to categorise 25 oyster species collected along the Chinese coast into four subgenera and they provided detailed descriptions of their morphological characteristics. Harry (1985) reported that, amongst the over 100 extant oyster species recorded worldwide, nearly two-thirds have synonymous names, suggesting the existence of taxonomic inconsistencies. Naming errors and synonymous names have frequently occurred in oyster taxonomy (Que et al. 2003), which has severely impacted the conservation of oyster genetic resources and the breeding of improved varieties. Molecular identification methods have significant advantages in taxonomy, as they can improve classification efficiency and accuracy. However, in practical production, their high cost prohibits non-destructive testing of large oyster populations.

In this study, we applied both traditional morphometrics and GM to analyse the morphology of *C.ariakensis* and *C.gigas*. Traditional morphometrics revealed differences in morphology between the two species. However, there was overlap between them, making it difficult to accurately identify them. GM can eliminate the effects of size, position and measurement angles by using TPS function analysis and PCA. It is a quantitative approach widely used to describe the shape of biological specimens and its covariation with other biological and environmental factors (Zelditch et al. 2004, Webster and Sheets 2010). Morphological variables are quantified using a set of Cartesian landmarks located on distinct homologous anatomical points and observed body shape variations are then displayed through user-friendly graphical representations (Adams et al. 2004, Zelditch et al. 2004, Mitteroecker and Gunz 2009). GM is a powerful technique capable of detecting even tiny morphological differences amongst groups of specimens (Mitteroecker and Gunz 2009, Webster and Sheets 2010).

Use of these techniques revealed the presence of shell shape differences between *C.ariakensis* and *C.gigas*. Discriminant analysis, based on the typical variables, resulted in a classification accuracy of 100% for the two oyster species. This demonstrates the feasibility of using GM for oyster morphology analysis and classification. Compared to molecular methods, GM offers advantages such as speed, non-destructive sampling and the ability to analyse large sample sizes in batches. Our results provide a theoretical foundation for the future application of GM in oyster classification and seedling breeding.

GM analysis of oysters needs to be based on a large number of specimens. It can be applied to analyse and compare the shape of oyster shells or other relevant structures and to assess the effects of environmental factors or genetic variations on shell shape. By collecting landmark coordinates on the shell, researchers can quantify and compare shape differences amongst different oyster populations or individuals. This information can provide insights into the genetic diversity and adaptive strategies of oysters in different environments. GM can also be used to study the ontogenetic changes in oyster shell shape. By capturing and analysing the shape variation at different growth stages, researchers can understand how the shell shape develops and changes during an oyster's lifespan. Overall, the application of GM in oysters can contribute to our understanding of the biology, evolution and ecological interactions of oysters, as it provides a quantitative and objective approach to studying shape variations, which can lead to valuable insights in oyster research and management.

## Supplementary Material

4BE54287-38B2-59D7-8D86-88F19DF9B4BF 10.3897/fmj.4.99103.suppl1 Supplementary material 1 Morph data of two oysters Data type Morphological data File: oo_923793.xlsx https://binary.pensoft.net/file/923793 Ying Tian

19588EB0-0677-5C1F-BB23-1313144E6906 10.3897/fmj.4.99103.suppl2 Supplementary material 2 The entire sequence of two groups of oysters Data type 16S rDNA sequence Brief description No1-No.3 are *Crassostreaariakensis* sequence No.4-No.6 are *Crassostreagigas* sequence. File: oo_923780.zip https://binary.pensoft.net/file/923780 Ying Tian

5520EF6D-F61E-53F4-8C9B-16DE493AE1DC 10.3897/BDJ.12.e115019.suppl3 Supplementary material 3 GM data Data type morphological data Brief description Landmark data for two groups of oysters. File: oo_923794.xlsx https://binary.pensoft.net/file/923794 Ying Tian

## Figures and Tables

**Table 1.** Morphological measurements of the two species of oysters using traditional morphometry.

Species	Statistic	Shell Height (mm)	Shell Length (mm)	Shell Width (mm)
*C.gigas*	Maximum	159.78	68.55	55.29
	Minimum	56.19	23.19	16.79
	Mean ± SD	94.92 ± 27.91	43.96 ± 11.15	29.33 ± 9.60
	Coefficient of Variation	29％	25％	33％
*C.ariakensis*	Maximum	205.64	114.53	39.57
	Minimum	78.49	26.28	11.12
	Mean ± SD	136.89 ± 32.95	69.01 ± 18.63	27.85 ± 8.17
	Coefficient of Variation	24.07％	26.99％	29.32％
